# Draft genome sequence of novel Candidatus *Ornithobacterium hominis* carrying antimicrobial resistance genes in Egypt

**DOI:** 10.1186/s12866-023-03172-6

**Published:** 2024-02-02

**Authors:** Nada Ahmed, Marwa Azab, Shymaa Enany, Amro Hanora

**Affiliations:** 1 Department of Microbiology and Immunology, Faculty of Pharmacy, Suez Canal University, Ismailia, Egypt; 2 Biomedical Research Department, Armed Force College of Medicine, Cairo, Egypt; 3 Department of Microbiology & Immunology, Faculty of Pharmacy, King Salman International University, Ras Sudr, Egypt

**Keywords:** *Ornithobacterium*, Nasopharyngeal microbiome, Antimicrobial resistance genes

## Abstract

**Background:**

*Candidatus Ornithobacterium hominis* (*O. hominis*), which was identified in nasopharyngeal swabs from Egypt, has been associated with respiratory disorders in humans. *O. hominis*, a recently identified member of the Flavobacteriaceae family, belongs to the largest family within the Bacteroidetes phylum. This family includes hundreds of species and 90 genera, including major human pathogens such as *Capnocytophaga canimorsus* and *Elizabethkingia meningoseptica*. Herein, we presented two draft genome assemblies of *O. hominis* that were extracted from metagenomic data using the Illumina sequencing method. The alignment of reads against the *O. hominis* genome was accomplished using BLASTN, and the reads with significant hits were extracted using Seqtk and assembled using SPAdes. The primary goal of this study was to obtain a more profound understanding of the genomic landscape of *O. hominis*, with an emphasis on identifying the associated virulence, antimicrobial genes, and distinct defense mechanisms to shed light on the potential role of *O. hominis *in human respiratory infections.

**Results:**

The genome size was estimated to be 1.84 Mb, including 1,931,660 base pairs (bp), with 1,837 predicted coding regions and a G+C content of 35.62%. Genes encoding gliding motility, antibiotic resistance (20 genes), and the toxA gene were all included in the genome assembly. Gliding motility lipoproteins (GldD, GldJ, GldN, and GldH) and the gliding motility-associated ABC transporter substrate-binding protein, which acts as a crucial virulence mechanism in Flavobacterium species, were identified. The genome contained unique genes encoding proteins, such as the ParE1 toxin that defend against the actions of quinolone and other antibiotics. The cobalt-zinc-cadmium resistance gene encoding the protein CzcB, which is necessary for metal resistance, urease regulation, and colonization, was also detected. Several multidrug resistance genes encoding proteins were identified, such as MexB, MdtK, YheI, and VanC.

**Conclusion:**

Our study focused on identifying virulence factors, and antimicrobial resistance genes present in the core genome of O. hominis. These findings provide valuable insights into the potential pathogenicity and antibiotic susceptibility of O. hominis.

**Supplementary Information:**

The online version contains supplementary material available at 10.1186/s12866-023-03172-6.

## Background

The nasopharynx serves as the primary ecological habitat for a significant portion of respiratory bacteria and can contribute to various middle ear and respiratory infections in humans. The ability of co-residing commensal nasopharyngeal bacteria to influence the behaviour of pathogenic species has been increasingly recognized. Thus, comprehending the interactions between commensal and pathogenic species that coexist in the nasopharynx is essential [1]. Studies on the nasopharyngeal microbiome have reported that it contains a wide range of previously unknown commensals, including the newly discovered bacterium *O. hominis.* Its closest relative is the avian respiratory pathogen *Ornithobacterium rhinotracheale* [2]. The exact role that *O. hominis* plays in disease emergence is still unknown. There is still much to learn about the biological functions of *O. hominis* and the molecular mechanisms by which it interacts with the host microbiota [2]. *O. hominis* is a bacterium that has never been cultured, but it has been found in nasopharyngeal microbiota sequencing data from a few laboratories worldwide [3]. *O. hominis* is a recently discovered member of the Flavobacteriaceae family, as confirmed by analysis of the 16S rRNA gene. This bacterium often colonizes the nasopharynx in infants. The genome contains genes that encode proteins, including antibiotic resistance proteins, competition factors, and a mitogenic toxin resembling that of *Pasteurella multocida* [3]. A PCR-based investigation determined the presence and persistence of *O. hominis* in the nasopharynx of a paediatric population at high risk of respiratory infection, and there has been an increase in interest in this bacterium [3]. Although its genomes may be determined via metagenomic data, isolating *O. hominis* strains is necessary to properly comprehend the role of the bacterium in human respiratory diseases [4].

Although its function in relation to ear and respiratory diseases is still unknown, *O. hominis* was first identified in infants from refugee camps in Thailand and Australia with high rates of respiratory diseases [5]. Children without a history of otitis media (OM) did not have *O. hominis* in their nasal microbiome, or it was present but with low relative abundance. The *Ornithobacterium was* most likely *O. hominis*, the only known species of humans in the genus *Ornithobacterium* that resides in the nasopharynx [6]. Children with OM often have *Ornithobacterium*, which may be a new otopathogen in this population [6]. Additionally*, Ornithobacterium, Helcococcus*, and *Dichelobacter* were found to be linked by network correlations and these bacteria could impact clinical outcomes. Recent research suggests that there may be novel bacterial species in genera that currently have no known human representatives, such as *Dichelobacter* and *Gracilibacteria,* or only one species, such as *Dolosigranulum,* within the nasal microbial ecosystem [6]. To gain a better understanding of the ecology and evolution of this important respiratory pathogen, the aim of this study was to provide the initial draft genome sequence of O. hominis from Egyptian populations. These data could help address the current knowledge gaps regarding the genomics of *O. hominis* and identify the virulence- and antimicrobial-related genes.

## Results

### Genome assembly

The assembled genome had a total length of 1,931,660 bp and an average G + C content of 35.62%, comprising 16 contigs, with an L50 count of 3 and an N50 length of 296,728 bp. The results of the Quast genome evaluation analysis are summarized in Additional file 1, which includes the quality assessment analysis statistics.

### Genome annotation

The taxonomic hierarchy for this genome was as follows: Cellular organisms > Bacteria > FCB group > Bacteroidetes/Chlorobi group > Bacteroidetes > Flavobacteriia > Flavobacteriales > Weeksellaceae > *Ornithobacterium* > *O. hominis*.

**Fig. 1:**
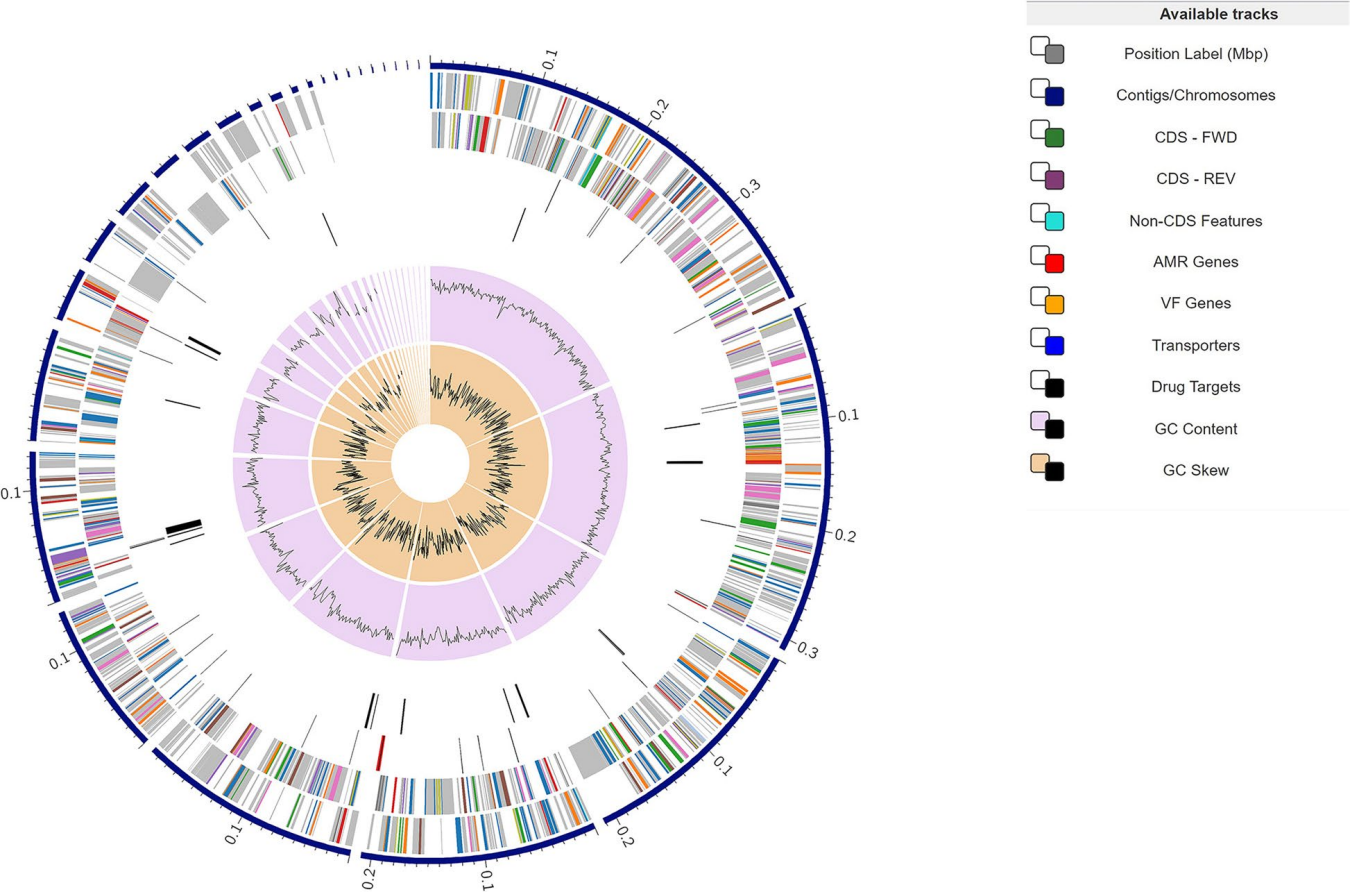
Genomic Annotations. Distribution of Genomic Annotations using circular genome viewer in BV-BRC (https://www.bv-brc.org/). This includes the contigs, CDS on the forward and reverse strands, RNA genes, CDS with homology to known antimicrobial resistance genes, CDS with homology to known virulence factors, GC content, and GC skew, in that order from outer to inner rings

This genome had 10 ribosomal RNA (rRNA) genes, 36 transfer RNA (tRNA) genes, and 1,837 protein-coding sequences (CDS). A total of 749 genes encoding hypothetical proteins and1,088 genes encoding proteins with assigned functions were included in the annotation. Of the proteins with functional assignments, 502 had Enzyme Commission (EC) numbers, 416 had Gene Ontology (GO) assignments, and 376 had KEGG pathway mappings. The genome had 0 genes encoding proteins that are members of genus-specific protein families (PLFams) and 1,193 genes encoding proteins that are members of cross-genus protein families (PGFams), according to the annotation results. This annotation covers two different types of protein families. The distribution of the genome annotations is shown in Fig. 1.

### Annotations with prokka

**Fig. 2:**
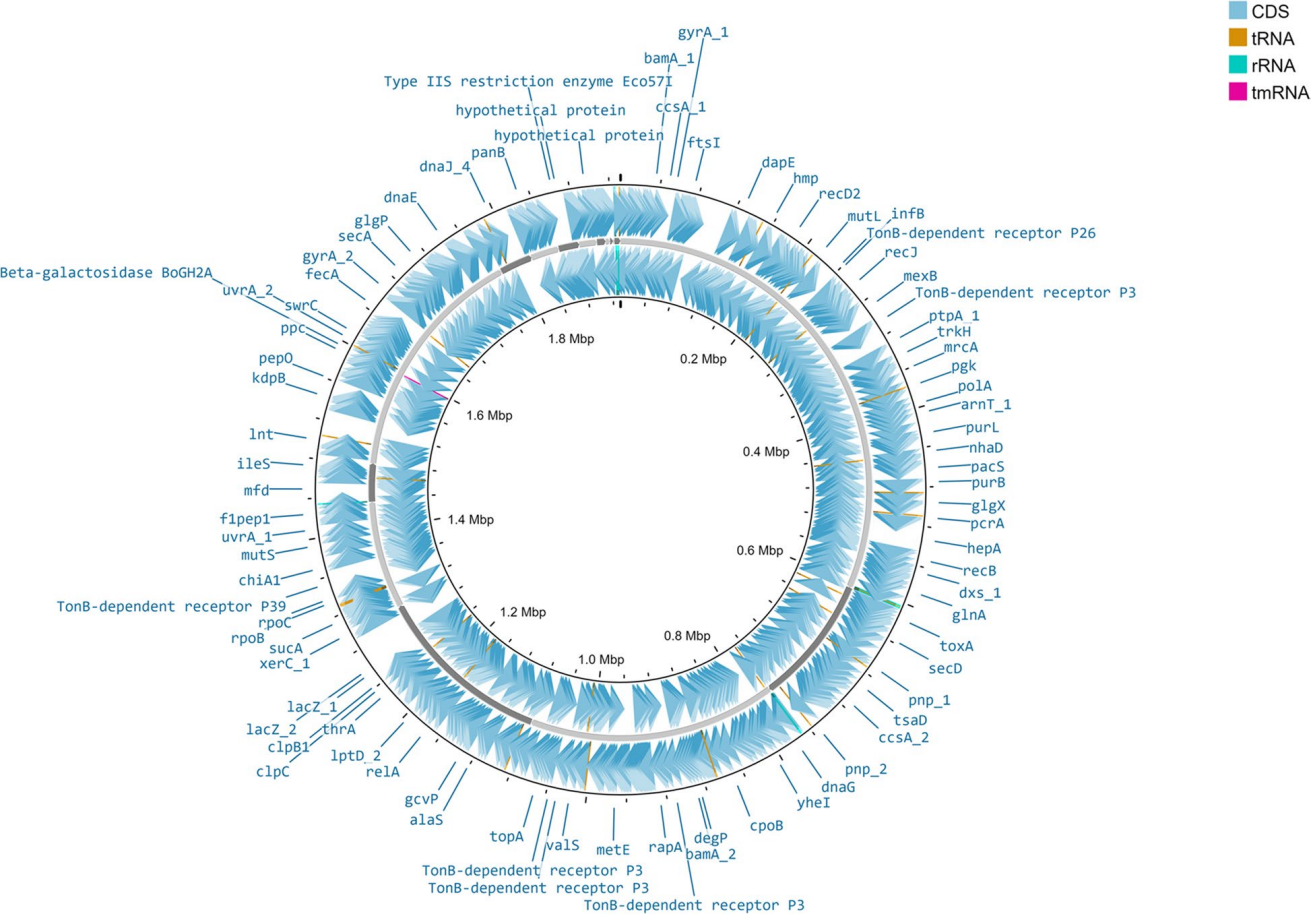
Prokka annotations. Circular representation showing Prokka functional annotations using Proksee. (https://proksee.ca/)

A total of 1791 genes were predicted using the Prokka pipeline. Of these, 1748 were protein-coding genes, 952 had nonhypothetical functions, and none of the genes had seed subsystem ontology. There were 477 genes with an EC number. The average protein length was 330 amino acids. A circular representation of the annotations of the draft genome MAGN1 is shown in Fig. 2. The annotations, which were performed by Pokka, include gliding motility lipoproteins (GldD), (GldJ), (GldN), and (GldH), and the swarming motility protein SwrC. The extracted genome contains encoding genes associated with virulence, including the dermonecrotic toxin *toxA*, the *parE1* toxin gene, the cobalt-zinc-cadmium resistance gene encoding protein CzcB, and the peroxide stress resistance gene encoding protein *yaaA*. Several multidrug resistance genes encoding proteins were identified, such as MexB, YheI, MdtK, MdtA, and VanC.

### Subsystem analysis

**Fig. 3:**
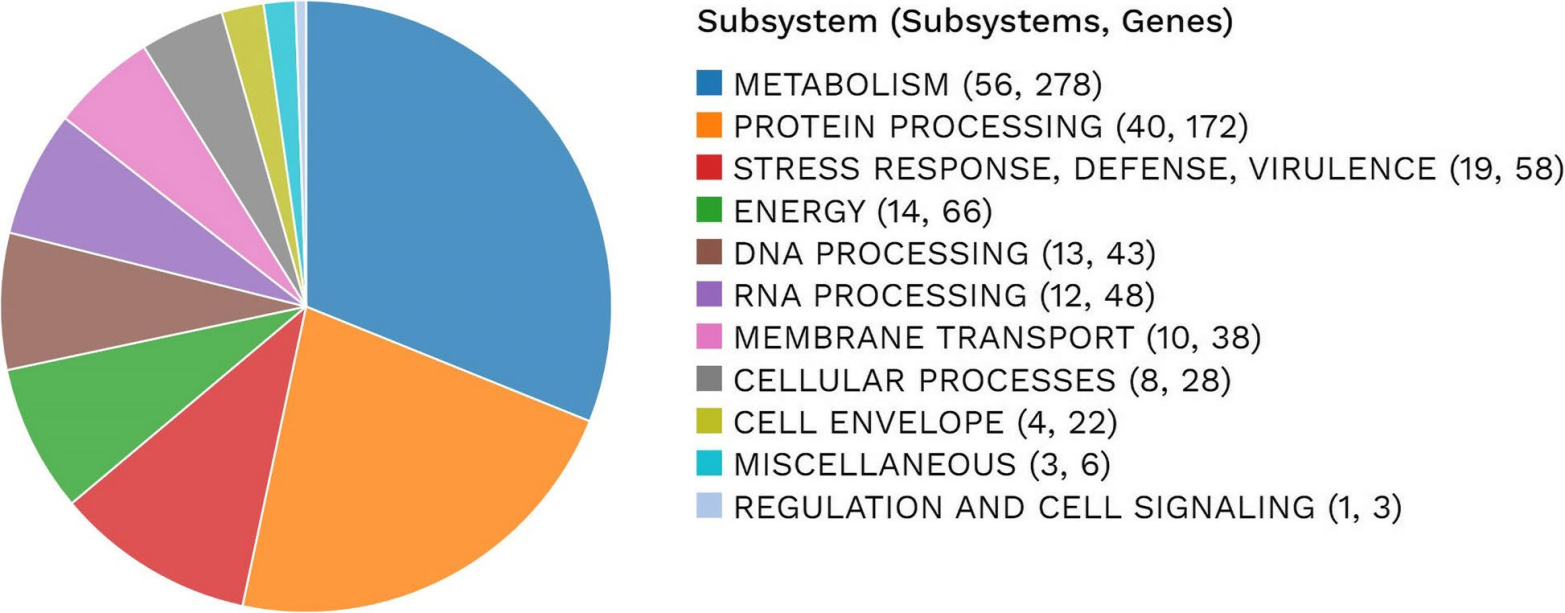
Subsystem Analysis. Pie chart representing the subsystem analysis for the MAGN1 draft genome

A subsystem, or a collection of related gene encoding proteins, collaborates to carry out a specific biological function or form a structural complex. A summary of the subsystems in the genome is shown in Fig. 3.

### Genes related to antimicrobial resistance

The extracted genome MAGN1 was found to have antimicrobial resistance genes such as *alr, ddl, ef-g, ef-tu, folA, dfr, folP, gyrA, gyrB, inhA, fabI, iso-tRNA, kasA, murA, rho, rpoB, rpoC, s10p*, and *s12p*. The proteins encoded by these genes play a role in resistance by altering the antibiotic target. The AMR mechanism of *gidB0* confers resistance through gene absence, while the AMR mechanism of *oxyR* regulates the expression of genes associated with antibiotic resistance. Table 1 (at the end of the document) lists the AMR genes that were identified in this genome, along with the associated classes of antibiotics, E-value, percent identity, and query coverage.

**Table 1:** Antimicrobial Resistance (AMR) Genes

Gene	Product	Antibiotic class	Antibiotics	Query coverage	Evalue	Percent identity	Accession
oxyR	Hydrogen peroxide-inducible genes activator	Isoniazid	Isoniazid	100%	0	100.00%	WP_119059582.1
folP	Dihydropteroate synthase (EC 2.5.1.15)	Sulfonamides	Sulfadiazine,sulfadimidine,sulfadoxine,sulfamethoxazole,sulfisoxazole,sulfacetamide,mafenide,sulfasalazine,sulfamethizole,dapsone	100%	0	99.30%	WP_119058480.1
Rho	Transcription termination factor Rho	Bicyclomycins	Bicyclomycins	100%	0	98.04%	WP_283670756.1
S12p	SSU ribosomal protein S12p (S23e)	Aminoglycosides	Streptomycin	100%	5.00E-83	100.00%	WP_119057766.1
gyrB	DNA gyrase subunit B (EC 5.99.1.3)	Clofazimine,gatifloxacin,ciprofloxacin	Ciprofloxacin	100%	0	99.54%	WP_119058897.1
folA, Dfr	Dihydrofolate reductase (EC 1.5.1.3)	Diaminopyrimidines	Trimethoprim,brodimoprim,tetroxoprim,iclaprim	100%	4.00E-114	98.77%	WP_119058034.1
inhA, fabI	Enoyl-[acyl-carrier-protein] reductase [NADH]	Isoniazid, Ethionamide, Triclosan	Isoniazid,ethionamide,triclosan	100%	0	99.64%	WP_119057337.1
S10p	SSU ribosomal protein S10p (S20e)	Tetracyclines, Glycylcyclines	Tetracycline,tigecycline	100%	2.00E-65	100.00%	WP_119057769.1
Iso-tRNA	Isoleucyl-tRNA synthetase (EC 6.1.1.5)	Mupirocin	Mupirocin (pseudomonic acid)	99%	0	100.00%	WP_119059585.1
gidB	16S rRNA (guanine(527)-N(7))-methyltransferase (EC 2.1.1.170)	Aminoglycosides	Streptomycin	100%	5.00E-142	97.57%	WP_119058444.1
EF-G	Translation elongation factor G	Fusidic acid	Fusidic acid	100%	0	99.86%	WP_119057768.1
MurA	UDP-N-acetylglucosamine 1-carboxyvinyltransferase (EC 2.5.1.7)	Fosfomycin	Fosfomycin	100%	0	100.00%	WP_119059381.1
rpoC	DNA-directed RNA polymerase beta' subunit (EC 2.7.7.6)	Myxopyronins,Corallopyronins, Peptide	Daptomycin	100%	0	99.51%	WP_283671279.1
Alr	Alanine racemase (EC 5.1.1.1)	Cycloserine	Cycloserine	100%	0	100.00%	WP_119058724.1
kasA	3-oxoacyl-[acyl-carrier-protein] synthase, KASII (EC 2.3.1.179)	Isoniazid, Triclosan	Isoniazid, Triclosan	100%	0	99.76%	WP_283671350.1
Ddl	D-alanine–D-alanine ligase (EC 6.3.2.4)	Cycloserine	Cycloserine	100%	0	99.69%	WP_283671268.1
EF-Tu	Translation elongation factor Tu	Elfamycins	Kirromycin,enacyloxin IIa,pulvomycin	100%	0	100.00%	WP_119058163.1
rpoB	DNA-directed RNA polymerase beta subunit (EC 2.7.7.6)	Rifamycins, Peptide antibiotics	Rifamycin,daptomycin,rifabutin,rifampin	100%	0	99.84%	SZD73303.1
gyrA	DNA gyrase subunit A (EC 5.99.1.3)	Fluoroquinolones Quinolones Quinolines	Clofazimine,ciprofloxacin,gatifloxacin,levofloxacin,moxifloxacin,nalidixic acid,ofloxacin,sparfloxacin,trovafloxacin	100%	0	99.64%	WP_283670346.1
	Subclass B1 beta-lactamase (EC 3.5.2.6)	Beta-lactam antibiotics	Beta-lactam antibiotics	100%	0	100%	WP_119059468.1

List of the AMR genes identified in the *O. hominis* draft genome, along with the antibiotic classes, query coverage, e-value, percent identity, and accession

### Phylogenetic analysis

**Fig. 4:**
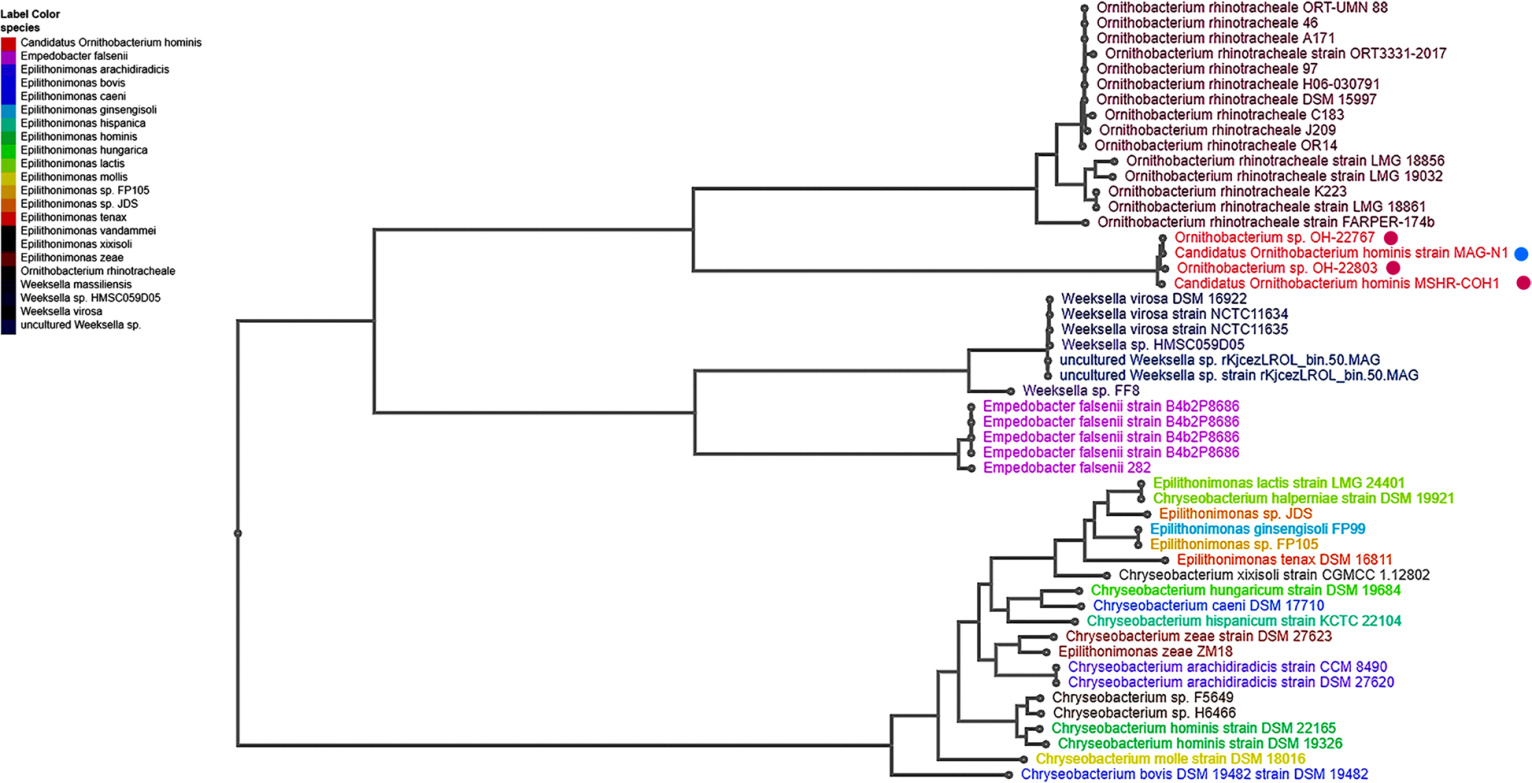
Phylogenetic analysis. RAxML phylogenetic tree for the draft genome of *O. hominis* and other species of *Flavobacteriacea.* The blue dot represents the draft genome of O. hominis, while the magenta dots indicate its closest relatives

The position of the *O. hominis* draft genome MAGN1 is illustrated in Fig. 4. The *O. hominis* draft genomes appear to be very closely related to *O. hominis* 22,767, as summarized in Table 2. The ANI between *O. hominis* MAGN1 and *O. hominis* 22,767 was 99.9%. The two genomes of *O. hominis* 22,767 and *O. hominis* 22,803 had an ANI of 98.5%, which is above the 96% threshold for strains of the same species.

**Table 2:** Pairwise comparison of the representative genomes 22767, 22803, and the MAGN1

Query	Reference	ANI Estimate	Matches	Total
Draft genome MAGN1	*O. hominis* 22803	98.4853	565	634
*O. hominis* 22767	*O. hominis* 22803	98.5183	566	632
*O. hominis* 22803	*O. hominis* 22767	98.5193	566	618
*O. hominis* 22803	Draft genome MAGN1	98.5212	566	618
Draft genome_MAGN1	*O. hominis* 22767	99.9591	628	634
*O. hominis* 22767	Draft genome MAGN1	99.9744	616	632

The results are sorted by ANI Match, with the highest match at the bottom

### ANI results with fast ANI

Investigation of the extracted MAGN1 genome revealed that the average nucleotide identity (ANI) with the representative *O. hominis* 22,767 genome was 99.97% (Table 2), which was the highest ANI. The ANI between MAGN1 and another commonly used representative genome, *O. hominis* -22,803, was 98.4%.

### Comparative genomics

**Fig. 5:**
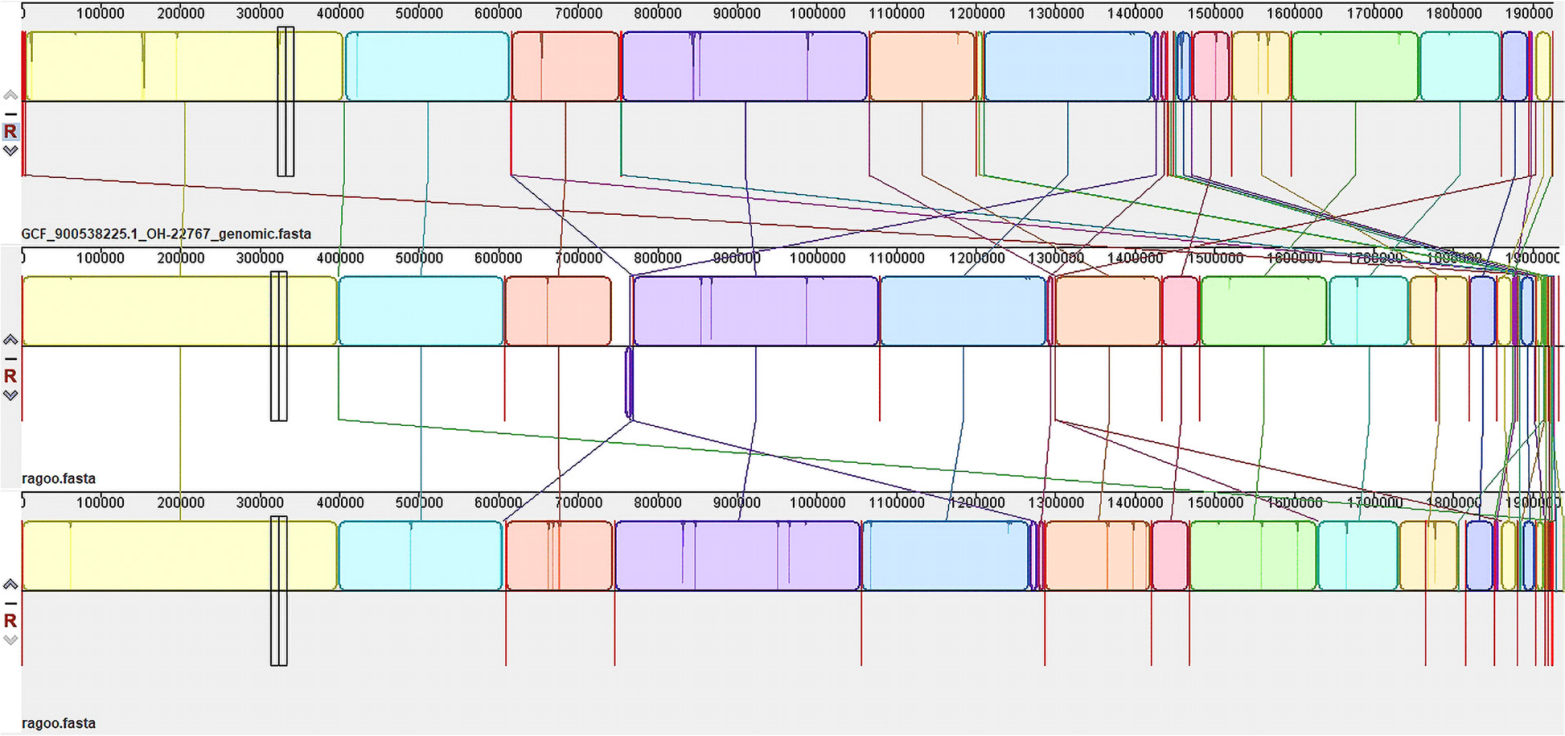
Comparative genomics**.** Genome alignment of the draft genome extracted from nasopharyngeal metagenomic samples against the *O. hominis* 22767 genome. The first genome is *O. hominis* 22767, while the second and third genomes are MAGN1 and MAGN2, respectively. Colored blocks indicate regions of sequence homology

Comparison of the *O. hominis* 22,767 genome and the draft genome MAGN1 was performed using MAUVE. The MAUVE alignment showed a high level of similarity between the *O. hominis* 22767 strain and the MAGN1 and MAGN2 draft genomes (Fig. 5).

### Pan genome

Pangenome analysis was performed on the two assemblies, MAGN1 and MAGN2, as well as the reference strains *O. hominis* 22767, *O. hominis* 22803, and the recently published MSHRCOH1 *O. hominis*. The total number of protein-coding genes was 8902; 8378 were in homologous families, and 524 were in singleton families. The total number of families was 2294 families. Of these, 1,770 gene families were in common in all genomes in the analysis, and 524 genes were singletons. Table 3 displays a summary of the statistics of the shared genes and pangenome datasets. A heatmap of comparative protein families summarizes the differences among all *O. hominis* strains. Information about the strains is summarized in Additional file 4.

**Table 3:** Pangenome analysis results

Genome	Genes	Number of Genes in Homologs	Number of Homolog Families	Number of Genes in Singletons
22767	1873	1678	1620	195
MAGN1	1823	1699	1680	124
MSHRCOH1	1746	1711	1682	35
22803	1712	1580	1561	132
MAGN2	1748	1710	1682	38

Summary statistics of the shared genes and pangenome analysis

### Core genome

The number of known core protein families was 1,446. There were several antimicrobial resistance-encoding genes in the core genome of *O. hominis.* Some virulence factors were also part of the core genome. Among the interesting genes in the core genome, the toxin-encoding genes *toxA* and *parE1* were identified, as well as genes associated with gliding motility. In related Flavobacterium species, the gliding motility lipoproteins (GldJ, GldD, and GldN) and the gliding motility-associated ABC transporter substrate-binding protein (GldA) were shown to play crucial roles in virulence [7]. There were genes encoding distinct proteins in the genome. These proteins include TraB, TraD, and TraA, which are predicted to be conjugative transposons. This suggests that mobile genetic elements play a potential role in the genome. Additionally, the resistance protein family ID (PGF_04493258) for cobalt-zinc-cadmium was detected.

However, no plasmid replicons were found. Unique genes encoding proteins, such as metallo beta-lactamases, the penicillin-binding protein (PBP) superfamily, class C-like beta-lactamases, and subclass B1 beta-lactamases, were present in the annotated genome and may have the ability to break down beta-lactam antibiotics. Urease accessory genes encoding the proteins UreE, UreF, UreG, and UreD were identified, along with those encoding the urease alpha subunit (EC 3.5.1.5), the urease beta subunit (EC 3.5.1.5), and the urease gamma subunit (EC 3.5.1.5).

### Accessory genome

The genes encoding hypothetical proteins make up approximately half of the MAGN1 draft genome. In addition, there is evidence of mobile elements, transfer- and mobilization-encoding genes, phages, and various lipopolysaccharide (LPS) synthesis clusters. A prophage region was identified, which appeared as short sections of less than 13 kb. The draft genome contained the cell wall-associated hydrolase family ID PGF_08065842. Additionally, genes encoding penicillin-binding proteins that confer multidrug resistance were identified. This genome also contained genes encoding unique proteins, such as ParA and ParB-related ThiF family proteins. These protein-coding genes were found to be conjugative transposons. The extracted genome MAGN1 had one pathogenic protein family that matched *Flavobacterium johnsoniae* UW101 with accession ID CP000685. This protein had 88% identity with the Bacteroides MobC/BfmC-like conjugative transposon protein (protein ID ABQ06040). Additionally, significant similarities were found between VapD (COG3309), a virulence-associated protein, and residues 1–91 in chain A of the immunoglobulin G-binding protein G. Specifically, the protein D PDB (PDB 3UI3_A) showed 51.69% identity (e-value 1e-24). The membrane protein insertion efficiency factor YidD is encoded by one of the genes exclusively found in the MAGN1 draft genome. The MAGN2 draft genome contained the RelK toxin-encoding gene, which is predicted to play a crucial role in antibiotic persistence. Virulence-associated genes that encoded protein domains were annotated as LbR_YadA-like domains (cd12820). This group includes virulence factors that have collagen-binding domains. In addition, the SpvB domain (PF03534) of the Salmonella virulence plasmid 65 kDa B protein and the BrkB virulence factor (PF03631) were also identified.

### Mobile genetic elements

In the MAGN1 genome, 43 mobile genetic elements (MGEs) were predicted using mobile OG-db. Seven of the MGEs integrate and excise while nineteen genes are linked to replication, recombination, and repair. The remaining encoding genes were predicted to be involved in stability, transfer, and defence (Additional file 2). One incomplete prophage region was identified. Regional details are summarized in Table 4. A representation of the mobile elements is provided in Additional file Fig. 5.

**Table 4:** Region details for the prophage sequence

**Region**	1
Region Length	10.3 Kb
Completeness (score)	Incomplete (30)
Region Position	161,604–171960
tRNA	0
Total Proteins	10
Phage Hit Proteins	7
Hypothetical Proteins	3
Phage + Hypothetical Protein %	100%
Bacterial Proteins	0
Attachment Site	No
Phage Species	7

prophage regions in the draft genome sequence of MAGN1 as predicted by PHASTER

### Proteome comparison

**Fig. 6:**
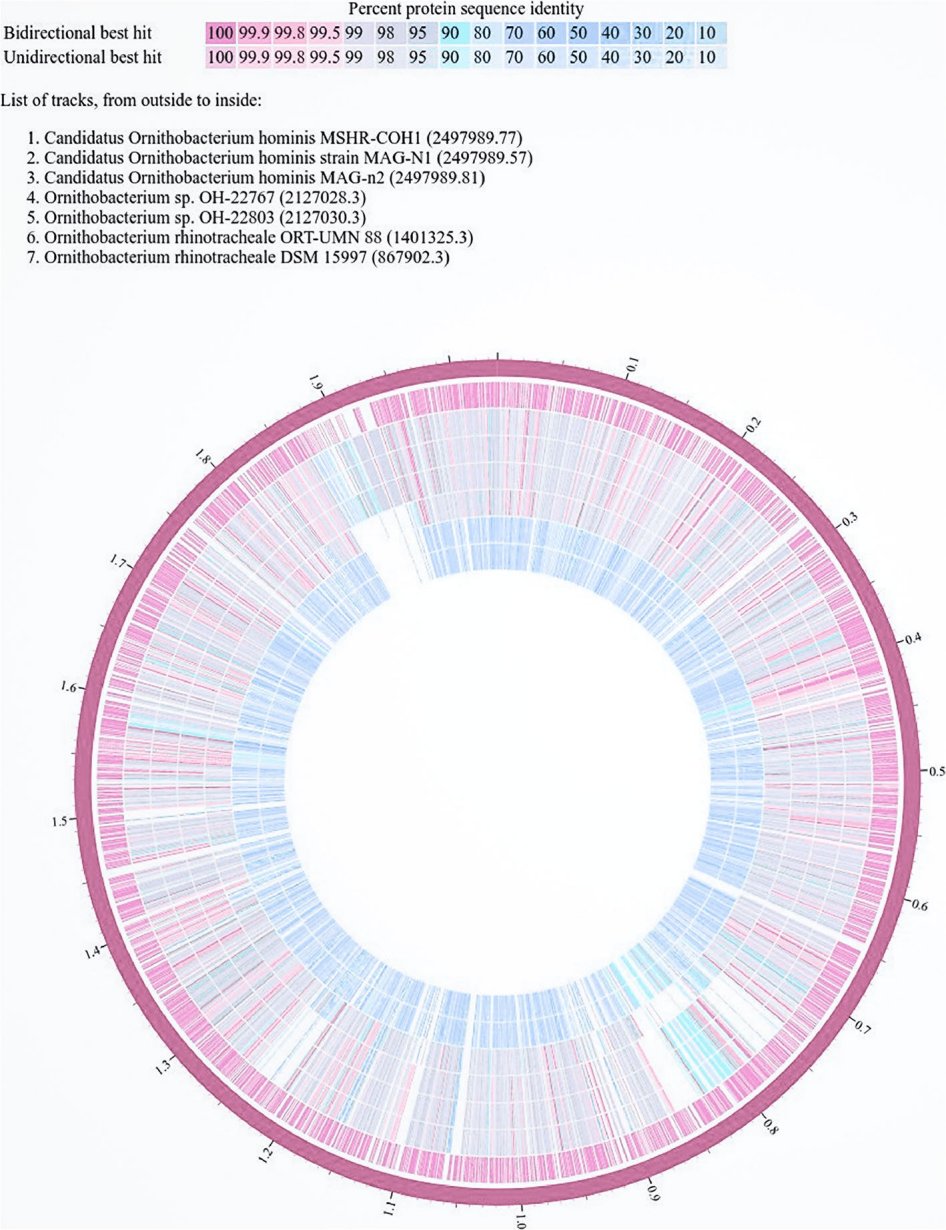
Genomic proteome analysis. Comparative genomic proteome analysis based on BLASTP generated by Circos. An illustration of the strengths of the BLAST hits (blue represents the strongest, red represents the weakest)

The extracted MAGN1, MAGN2, *Ornithobacterium* sp. *O. hominis* 22,767, Ornithobacterium sp. *O. hominis* 22,803, *Ornithobacterium rhinotracheale* ORT-UMN 88, *Ornithobacterium rhinotracheale* DSM 15997, and *Candidatus Ornithobacterium hominis* MSHR-COH1 genomes were compared using the proteome comparison service in the BV BRC tool to identify insertions and deletions. The sequence-based comparison tool that was a component of RAST originally served as the foundation for the Proteome Comparison service. Utilizing BLASTP and setting the parameters to a minimum coverage of 30%, a minimum identity of 10% and a BLAST E-value of 1e-5, this tool used protein similarity to assign each gene a colour and, when compared to the reference genome, designated each gene as either unique, a unidirectional best hit, or a bidirectional best hit. The proteome comparison is shown in Fig. 6 and is represented in a circular graphical format. The two strains of *O. rhinotracheale* were chosen because they are closely related to the *O. hominis* genome. All *O. hominis* strains had 100% identity for the multiple antibiotic resistance protein MarC. However, only 49% identity was established among *O. rhinotracheal* strains. GldD, GldJ, GldN, and the substrate-binding protein GldG, which is associated with the gliding motility-associated ABC transporter, were present in all variants of *Ornithobacterium*. The percent identity for these gene-encoding proteins was 98%, 99%, 99%, and 99%, respectively, compared to *O. rhinotracheal* strains. The percent identity for ORT-UMN 88 and DSM 15997 was lower, at 60%, 74%, 71%, and 49%, respectively. The type III restriction-modification system methylation subunit (EC 2.1.1.72), the putative type II restriction endonuclease, the Type III restriction enzyme, the res subunit: DEAD/DEAH box helicase, the N-terminal, and the Type III restriction-modification system methylation subunit (EC 2.1.1.72) were found only among *Candidatus Ornithobacterium hominis* MSHR-COH1. The details of the comparative proteome analysis are summarized in Additional file 3. The urease alpha, beta, and gamma subunits were present in all *O. hominis* genomes with 100% percent identity. Urease accessory genes encoding the proteins UreD, UreG, UreE, and UreF were found in all variants of *O. hominis*, with percent identities of 96%, 97%, 100%, and 100%, respectively.

### Protein modelling

**Fig. 7:**
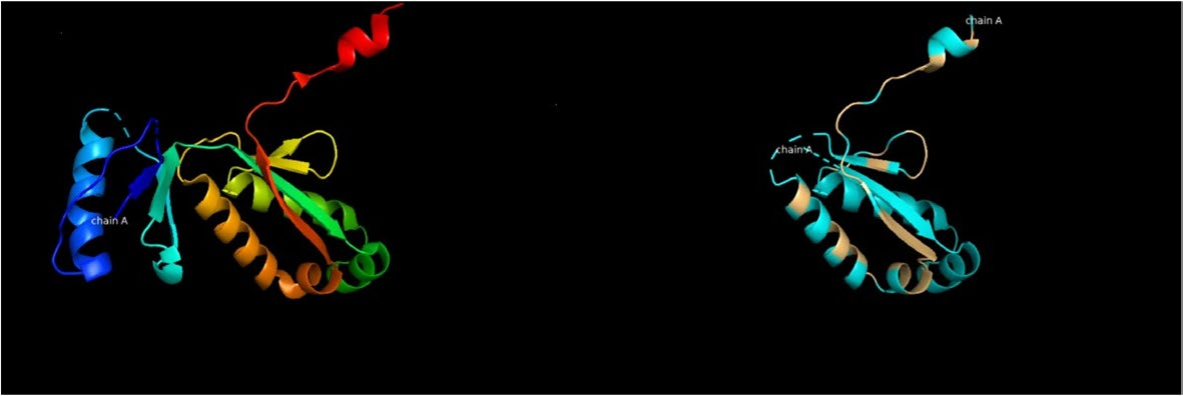
Protein modeling. Structure of 3UI3_A and a model of the equivalent region of *O. hominis* vapD. The 3ui3 structure (left) is colored with a rainbow spectrum to indicate position. The *O. hominis* model (right) is colored according to amino acid identity to 3UI3_A, with identical residues in light orange and nonidentical residues in blue

The virulence-associated protein in the *O. hominis* genome was found to have a significant 51.69% similarity with residues 1–91 of chain A of immunoglobulin G-binding protein G, virulence-associated protein D (PDB ID: 3UI3_A). The configuration of 3UI3_A and the corresponding model of the vapD protein associated with *O. hominis* are shown in Fig. 7. VapD has recently been linked to the Cas2 family of ribonucleases, which are connected to the CRISPR system of microbial immunity. Additionally, the predicted structures were remarkably similar.

## Discussion

Children in Egypt are often prescribed beta-lactam antibiotics, which can disrupt their microbiomes due to the widespread use of empirical antibiotic therapy. The extracted genome MAGN1 includes unique genes encoding proteins, such as metallo-beta-lactamases (MBLs). MBLs can hydrolyse carbapenems despite their seemingly low abundance [7]. MBLs are a common mechanism of resistance in carbapenem-resistant enterobacteria (CRE), as well as in carbapenem-resistant nonfermentative bacteria such as *Pseudomonas aeruginosa* and *Acinetobacter baumannii.*

Antimicrobial resistance genes were found in the extracted genome, such as the *murA* gene, which is responsible for conferring fosfomycin resistance [8]. Some of the encoded genes are genetic determinants for aminoglycosides, such as *gidB*, and fluoroquinolones, such as *gyrA*. In this study, the methyltransferase gene *gidB* was found, which has been identified as a significant contributor to antibiotic resistance in gram-negative bacteria such as salmonella [9]. Furthermore, the *gidB* protein plays a significant role in altering susceptibility to antibiotics such as neomycin and streptomycin [9]. Additionally, its absence confers resistance to these antibiotics [9]. The draft genome also included a transcriptional regulator gene, *oxyR*. By decomposing hydrogen peroxide (H2O2) produced by the host defence response, *oxyR* functions as a transcriptional regulator in many bacteria and promotes infection [10]. Previous research has shown that *oxyR* plays a crucial role in controlling the development of biofilms, cell motility, the expression of pilus-related genes, the production of surface polysaccharides, and mucosal colonization [10]. In another study, *oxyR* was identified as a transcriptional regulator of the H2O2 stress response that affects the growth of *Acinetobacter baumannii* strains during H2O2 exposure. *OxyR* controls the *katE* and *ahpF1* genes that encode enzymes that degrade H2O2. Inactivating *oxyR* impedes the growth of both antimicrobial-susceptible and multidrug-resistant *Acinetobacter baumannii* strains. Additionally, an *oxyR* mutant showed reduced fitness during lung infection in murine lungs [11].

The use of antibiotics poses a significant environmental threat to pathogenic bacteria, as they are frequently exposed to them. Previous research has shown that antibiotics can induce oxidative stress in bacterial cells [12]. The role of *oxyR* in modulating antibiotic resistance has been reported in earlier investigations [13]. Srinivasan et al. 2013 presented experimental evidence supporting the role of *oxyR* in conferring resistance to gastrointestinal stresses and antimicrobial agents within the hypervirulent K1 serotype of *Klebsiella pneumoniae* NTUH-K2044. Srinivasan et al. 2013 examined how *oxyR* affects antibiotic resistance and revealed that the absence of this protein increases susceptibility to a variety of antibiotics. In the case of *Klebsiella pneumoniae* NTUH-K2044, the deletion of *oxyR* diminished both drug extrusion capability and the expression levels of efflux pumps, indicating the potential regulatory role of *oxyR* in governing the expression of these genes. Furthermore, in this study, researchers found that *oxyR* contributes to resistance against chlorhexidine and benzalkonium chloride, and *oxyR* reduces the ability to of the host to eliminate the nematode *Caenorhabditis elegans*, thereby emphasizing its critical involvement in virulence [14]. Rel toxin encoding genes found in the MAGN2 assembly were found to be elevated in *Mycobacterium tuberculosis* in response to rifampin (*relE, relG*,and *relK)*, gentamicin (*relG* and *relK*), and levofloxacin (*relG* and *relK*). Each of these genes plays a unique role in antibiotic persistence and increased survival [15].

In addition to the identified antimicrobial resistance encoding genes, the extracted genome of *O. hominis* had mobile encoding genes that confer resistance to heavy metal compounds. The extracted genome revealed the presence of the cobalt-zinc-cadmium resistance gene encoding the protein CzcB, which is essential for metal resistance, urease regulation, and colonization. In a recent study [16], the authors discussed the potential coselection of heavy metal tolerance and antimicrobial resistance. The two heavy metals most frequently found to be linked to antimicrobial resistance are zinc and cadmium. In the extracted draft genomes, specific virulence factors that may contribute to the spread and virulence potential of the strain *O. hominis* have been identified, including VapD, a protein associated with virulence. The mechanism by which VapD confers pathogenicity is not well understood. The expression of the VapD protein is increased during biofilm formation in *Xylella fastidiosa* [17]. The draft genome MAGN1 was found to contain the ParE toxin-encoding gene, indicating that the toxin ParEI likely targets DNA gyrase and is involved in a RecA-dependent SOS response. This is similar to the ParE1 and ParE2 toxins found in *Vibrio cholerae* chromosome II and the CcdB toxin found in the *Escherichia coli* F-plasmid [18]. To fully understand the mechanism of ParDEI-mediated tolerance, in-depth mechanistic studies are needed. However, it is anticipated that this process would resemble CcdB toxin-mediated antibiotic tolerance [19]. It is hypothesized that the amount of Lon and/or other proteases rises in conditions of antibiotic stress leading to the degradation of the ParDI antitoxin and the release of the ParEI toxin-antitoxin complex [18]. To cause DNA damage and trigger the SOS reaction, DNA gyrase and free toxin interact. This activation of additional cellular systems is involved in persister cell formation [18]. Our findings reveal that the extracted genome contains the *brkB* gene, which encodes a protein domain associated with virulence. *BrkB*, a virulence factor, is crucial for resisting complement-dependent killing by serum [20]. Specific virulence- and antibiotic resistance-encoding genes were found in this genome. These genes may provide insights into the various strategies employed by this fastidious bacterium to effectively colonize the respiratory tract, evade host immune responses, and survive antimicrobial treatment. In microbial ecosystems, hydrogen peroxide is frequently produced due to chemical oxidation or host defence mechanisms. Hydrogen peroxide can cross membrane barriers and damage important intracellular macromolecules, such as DNA and iron-dependent enzymes [21]. Bacteria activate a regulon to employ a range of defensive mechanisms to safeguard themselves against the harmful effects of hydrogen peroxide. A crucial component of the H_2_O_2_ stress response is *yaaA* [21]. The draft genome MAGN1 was found to contain the gene *yaaA.* There are many *yaaA* genes in bacteria, including in anaerobes and aerobes. Even anaerobes are occasionally exposed to oxygenated fluids and typically have genes encoding antioxidant enzymes, including H_2_O_2_ scavengers. Therefore, the presence of these genes in anaerobes does not necessarily imply that they have a nonoxidative stress role. *Lactobacillus gasseri*, a bacterium that utilizes hydrogen peroxide to inhibit competitors, possesses a *yaaA* gene that can be identified [22]. Further studies must be conducted to investigate the role of the *yaaA* gene in *O. hominis* and its correlation with the nasal microbiota.

The emergence of *O. hominis* as a potential cause of respiratory infections in humans has led to significant concerns within the field of infectious diseases. These infections emphasize the importance of conducting thorough research into pathogenic mechanisms, host interactions, and resulting implications for public health. However, *O. hominis* may be a commensal bacterium because it has also been found in the respiratory tracts of healthy humans who do not exhibit any clinical signs of infection. This indicates that the presence of *O. hominis* does not always result in clinical disease; although, it is still unclear whether the same strain of *O. hominis* can exist as a commensal and become pathogenic under certain conditions.

Gaining phenotypic information on the antimicrobial resistance of *O. hominis* will require further investigation. Thus, the hypothesized antimicrobial resistance is not an exact reflection of the actual antimicrobial resistance but rather indicative of potential resistance. Comparing and contrasting genotypes and phenotypes to identify similarities and differences, as well as developing minimum inhibitory concentration limits for *O. hominis*, will require further research. Long-read sequencing technologies, such as Oxford Nanopore sequencing, may also be helpful in completing these genomes in future studies.

## Conclusion

The draft genome of *O. hominis* revealed the presence of multiple genes responsible for encoding virulence factors, including *toxA* and gliding motility genes, as well as genes encoding antibiotic resistance. Among the virulence factors identified in the retrieved genomes, VapD, which could impact the spread of *O. hominis*. The genome also contained gliding motility lipoproteins and a toxin component of the Txe-Axe toxin-antitoxin module. In addition, the genome contained genes encoding unique proteins, such as class C-like beta-lactamases and type 2 metallo-beta-lactamases, which can hydrolyse beta-lactam antibiotics. The genome sequence of *O. hominis* significantly enhances our understanding of the genetic structure of this bacterium, paves the way for more in-depth studies, and provides a valuable resource for future investigations into the potential role of *O. hominis* in respiratory diseases. However, additional analysis and experimentation are needed to identify the genetic basis of virulence in this bacterium. Future studies should investigate the function of numerous genes encoding hypothetical proteins in the *O. hominis* genome. Additionally, further research is needed to determine the phenotypic information regarding *O. hominis* 's resistance to antibiotics.

## Materials and methods

### Ethics approval

This study was approved by the Faculty of Pharmacy, Suez Canal University ethical board (No. 202012PHD4). All experiments were performed in accordance with relevant guidelines and regulations. Informed written consent was obtained from all adult subjects and the parents of every child recruited for the purposes of screening, enrolment, and specimen collection in this study.

### Specimen collection methods

The collection of samples was conducted from November 2021 to April 2022 and from December 2022 to March 2023. Nasopharyngeal samples were collected from 10 infants (ranging from 0–24 months of age [3]) who were being investigated for pneumonia, as well as from 10 healthy infants. An aseptic approach was used to collect all specimens. Specimens were collected from the posterior wall of the nasopharynx using Catch-AllTM sample collection swabs soaked in a sterile solution of 0.15 M NaCl with 0.1% Tween 20. The samples were then immediately placed in STGG (skim milk, tryptone, glucose, and glycerol) storage media and frozen at -80 °C until DNA extraction was conducted.

### DNA extraction

To disperse the bacterial entrapment, the collection tubes were vortexed slowly. The DNeasy PowerSoil Pro DNA Isolation Kit (Qiagen, Valencia, CA), catalogue number 47016–250, was used to efficiently extract genomic DNA from the swabs following the manufacturer’s instructions.

### PCR amplification and shotgun metagenomic sequencing

#### PCR optimization

Immediately after DNA extraction, PowerUp SYBR Green master mix was used to conduct qPCRs with 20 μL reaction volumes. The forward primer 5'CTTATCGGGAGGATAGCCCG-3' and the reverse primer 5'GAAGTTCTTCACCCCGAAAACG-3' were used in the 16S rRNA gene screen to specifically target the V2-V5 region and yield a 700 bp product. Amplification was performed under the following conditions: 94 °C for 5 min, followed by 40 cycles of 94 °C for 30 s, 53 °C for 30 s, and 68 °C for 1 min. Finally, a melting curve analysis was conducted. The cycle threshold (Ct) was less than 40, and the peak melting temperature (Tm) was between 80 and 86. PCR was performed on a negative control sample to eliminate the possibility of contamination.

### Shotgun metagenomic sequencing

Utilizing MiSeq Reporter v2.3 (Illumina) according to the manufacturer’s metagenomics workflow, shotgun metagenomic sequencing was carried out on all samples at IGA Technology Services (Udine, Italy) with a coverage of 10X. The Agilent 2100 Bioanalyzer High Sensitivity DNA assay (Agilent Technologies, Santa Clara, CA) was used to evaluate the quality of both the input and the final libraries after they had been analysed using the Qubit 2.0 Fluorometer (Invitrogen, Carlsbad, CA). The demultiplexing of sequences was based on index sequences (also called barcodes).

### Sequence preprocessing and analysis

The Illumina platform generates two reads for forward and reverse sequencing. Quality filtering of sequence data was performed using FASTQC v0.12.0 [23]. The software Trimmomatic v0.39 was used to trim low-quality sequences [24]. Bowtie2 v2.3.2 was utilized to exclude human reads [25]. This was achieved by downloading the host human genome database, building a database for the host genome, mapping reads against the host, converting the SAM file to a BAM file, extracting the unmapped reads, sorting the BAM file to organize paired reads, and finally converting BAM to fastq. Taxonomic profiling for metagenomic samples was performed using Kaiju v1.9.2 [26].

### Genome extraction from metagenomic samples

Kraken v0.10.6 was used to initially categorize raw reads [27]. Bowtie2 v2.3.2 was used to exclude reads identified as human reads. Using BLASTN, the remaining reads were aligned against the representative *O. hominis* genome. The reads that had statistically significant hits were extracted using the Seqtk tool v0.5.0 [28] and assembled using SPAdes v3.11.1 [29]. The data were downloaded from Linux. The samples were then reciprocally examined using BLAST + to identify additional contigs that were present in all runs. The curated contig sets were manually improved using Ragoo v1.1 [30]. Using Minimap2 alignments, an open-source command-line program for Python was utilized to organize and align the contigs of a genome assembly with a single reference genome [30]. The quality of the genome was evaluated using the QUAST v 5.2.0 assembly assessment [31]. The RAST Tool Kit v1.073 (RASTtk) was used at BV-BRC to annotate curated contigs [32] and Prokka [33]. Using RAxML v8, phylogenetic trees were created [34]. Genome alignment of our draft genome to the already published representative *O. hominis* was performed using Mauve v2.3.1 [35]. Using PATRIC, antimicrobial resistance encoding genes were found [32]. Mobile genetic elements were predicted using Proksee [36]. Phage sequences were identified using Phaster [37]. Utilizing Fast ANI, an ANI analysis was carried out [38]. The representative genomes *O. hominis* 22,767, *O. hominis* 22,803, and MRSHR-COH1 were used to compare the genome sequences produced in this investigation. Pangenome analysis was conducted in Kbase [39] utilizing the "build Pangenome with OrthoMCL" tool. The construction of the *O. hominis* pangenome was performed by the OrthoMCL algorithm [40]. Scoary v1.6.16 was used to compare the genetic content of the *O. hominis* genomes [41]. Genes unique to each group were further characterized by comparing the relevant amino acid sequences to the BLAST protein database. A minimum identity percentage of 90% and a minimum coverage percentage of 50% were necessary for accurate identification.

### Protein modelling

Using the program Fugue version 2.0 [42], the sequence of the virulence-associated protein in *O. hominis* was searched against a database of all chains in the Protein Data Bank (PDB). Significant similarity with 51.69% identity was found for residues 1–91 in chain A of immunoglobulin G-binding protein G, virulence-associated protein D PDB (PDB ID: 3UI3_A). Models were created using the "very slow" refining method in Modeller 10.4 [43]. PyMOL Molecular Graphics System v2.5 utilized for analysis.

### Supplementary Information

**Additional file 1. **Results for Quast Genome Assessment Analysis.

**Additional file 2. **List of the mobile genetic elements in the draft genome.

**Additional file 3. **Protein comparison among *O. hominis *genomes and *O. rhinotracheal* strains using BLASTP.

**Additional file 4. **Comparative proteome analysis heatmap.

**Additional file 5. **Mobile elements representation.

## Data Availability

The datasets supporting the conclusions of this article are available in the National Centre for Biotechnology Information (NCBI) GenBank with accession number JAODHC000000000 ( https://www.ncbi.nlm.nih.gov/datasets/genome/GCA_025367715.1/) [44] and MAGN2 under accession number JASJOL000000000 ( https://www.ncbi.nlm.nih.gov/datasets/genome/GCA_030149645.1/) [45]. Sequence information was made available to the NCBI BioProject (www.ncbi.nlm.nih.gov/bioproject) with an accession number. PRJNA911048 and Biosample number SAMN30901436. Patient and sample metadata were submitted to DB Biosample with the accession numbers SAMN32164034 to SAMN32164038 and SAMN30864084.
