# Supplementary material for: Draft genome sequence of novel Candidatus Ornithobacterium hominis carrying antimicrobial resistance genes in Egypt
Source: BMC Microbiol. 2024 Feb 2;24:47. doi: 10.1186/s12866-023-03172-6 (PMC10835994; doi:10.1186/s12866-023-03172-6)
Supplement: Supplementary file 1 — Additional file 1. Results for Quast Genome Assessment Analysis. [file 12866_2023_3172_MOESM1_ESM.docx]

**Additional file 1**

**Additional file 1: Results for Quast Genome Assessment Analysis**

| **Assembly** | **Draft Genome** |
| --- | --- |
| Contigs (> = 0 bp) | 16 |
| Contigs (> = 1000 bp) | 16 |
| Contigs (> = 5000 bp) | 13 |
| Contigs (> = 10000 bp) | 13 |
| Contigs (> = 25000 bp) | 10 |
| Contigs (> = 50000 bp) | 7 |
| Total length (> = 0 bp) | 1931660 |
| Total length (> = 1000 bp) | 1931660 |
| Total length (> = 5000 bp) | 1928111 |
| Total length (> = 10000 bp) | 1928111 |
| Total length (> = 25000 bp) | 1879904 |
| Total length (> = 50000 bp) | 1768145 |
| Contigs | 16 |
| Largest contig | 608433 |
| Total length | 1938509 |
| Reference length | 1924273 |
| GC (%) | 35.62 |
| Reference GC (%) | 35.59 |
| N50 | 296728 |
| NG50 | 296728 |
| N90 | 50592 |
| NG90 | 50592 |
| AuN | 336381.8 |
| AuNG | 338870.4 |
| L50 | 3 |
| LG50 | 3 |
| L90 | 7 |
| LG90 | 7 |
| Ns per 100 kbp | 159.92 |

**N50 is the shortest contig's sequencing length at 50% of the entire assembly length, NG50 is the length at which at least half of the reference genome is covered by the collection of all contigs that are that length or longer. The areas under the Nx and NGx curves are denoted as auN and auNG. L50 is the bare minimum of contigs required to cover 50% of the assembly.**
