# Supplementary material for: Draft genome sequence of novel Candidatus Ornithobacterium hominis carrying antimicrobial resistance genes in Egypt
Source: BMC Microbiol. 2024 Feb 2;24:47. doi: 10.1186/s12866-023-03172-6 (PMC10835994; doi:10.1186/s12866-023-03172-6)
Supplement: Supplementary file 2 — Additional file 2. List of the mobile genetic elements in the draft genome. [file 12866_2023_3172_MOESM2_ESM.docx]

**Additional file 2: List of the mobile genetic elements in the draft genome**

**GPD is the GutPhage Database.**
