# Supplementary material for: Draft genome sequence of novel Candidatus Ornithobacterium hominis carrying antimicrobial resistance genes in Egypt
Source: BMC Microbiol. 2024 Feb 2;24:47. doi: 10.1186/s12866-023-03172-6 (PMC10835994; doi:10.1186/s12866-023-03172-6)
Supplement: Supplementary file 3 — Additional file 3. Protein comparison among O. hominis genomes and O. rhinotracheal strains using BLASTP. [file 12866_2023_3172_MOESM3_ESM.docx]

**Additional file 3: Protein comparison among *O. hominis* genomes and *O. rhinotracheal* strains using BLASTP.**

**The genomic name abbreviation of each genome is highlighted in bold.**
