# Supplementary material for: Draft genome sequence of novel Candidatus Ornithobacterium hominis carrying antimicrobial resistance genes in Egypt
Source: BMC Microbiol. 2024 Feb 2;24:47. doi: 10.1186/s12866-023-03172-6 (PMC10835994; doi:10.1186/s12866-023-03172-6)
Supplement: Supplementary file 4 — Additional file 4. Comparative proteome analysis heatmap. [file 12866_2023_3172_MOESM4_ESM.docx]

**Additional files figures**

**Additional file 4**

**title:** Comparative proteome analysis heatmap


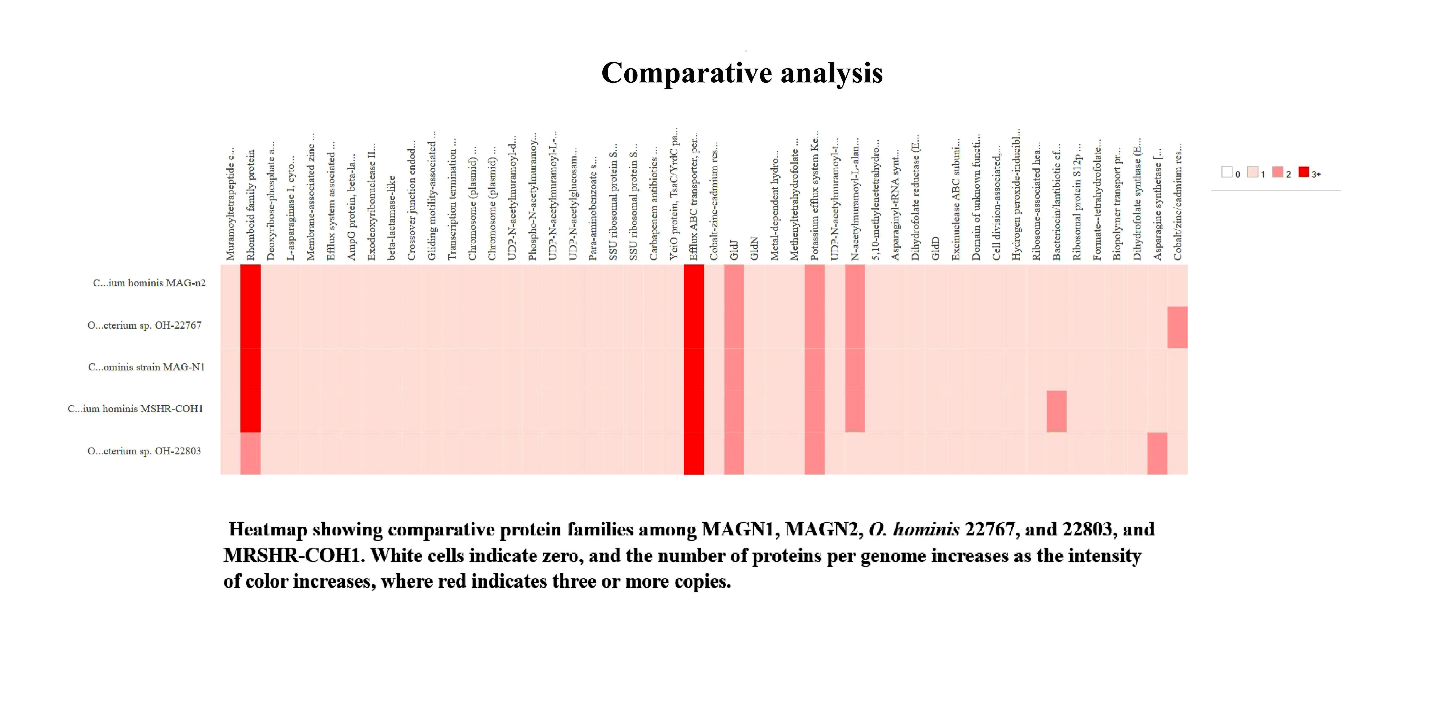


**legend:** Heatmap showing comparative protein families among MAGN1, MAGN2, *O. hominis* 22767, and 22803, and MRSHR-COH1. White cells indicate zero, and the number of proteins per genome increases as the intensity of color increases, where red indicates three or more copies.
