# Supplementary material for: Draft genome sequence of novel Candidatus Ornithobacterium hominis carrying antimicrobial resistance genes in Egypt
Source: BMC Microbiol. 2024 Feb 2;24:47. doi: 10.1186/s12866-023-03172-6 (PMC10835994; doi:10.1186/s12866-023-03172-6)
Supplement: Supplementary file 5 — Additional file 5. Mobile elements representation. [file 12866_2023_3172_MOESM5_ESM.docx]

**Additional File 5**

**Title:** Mobile elements representation**.**


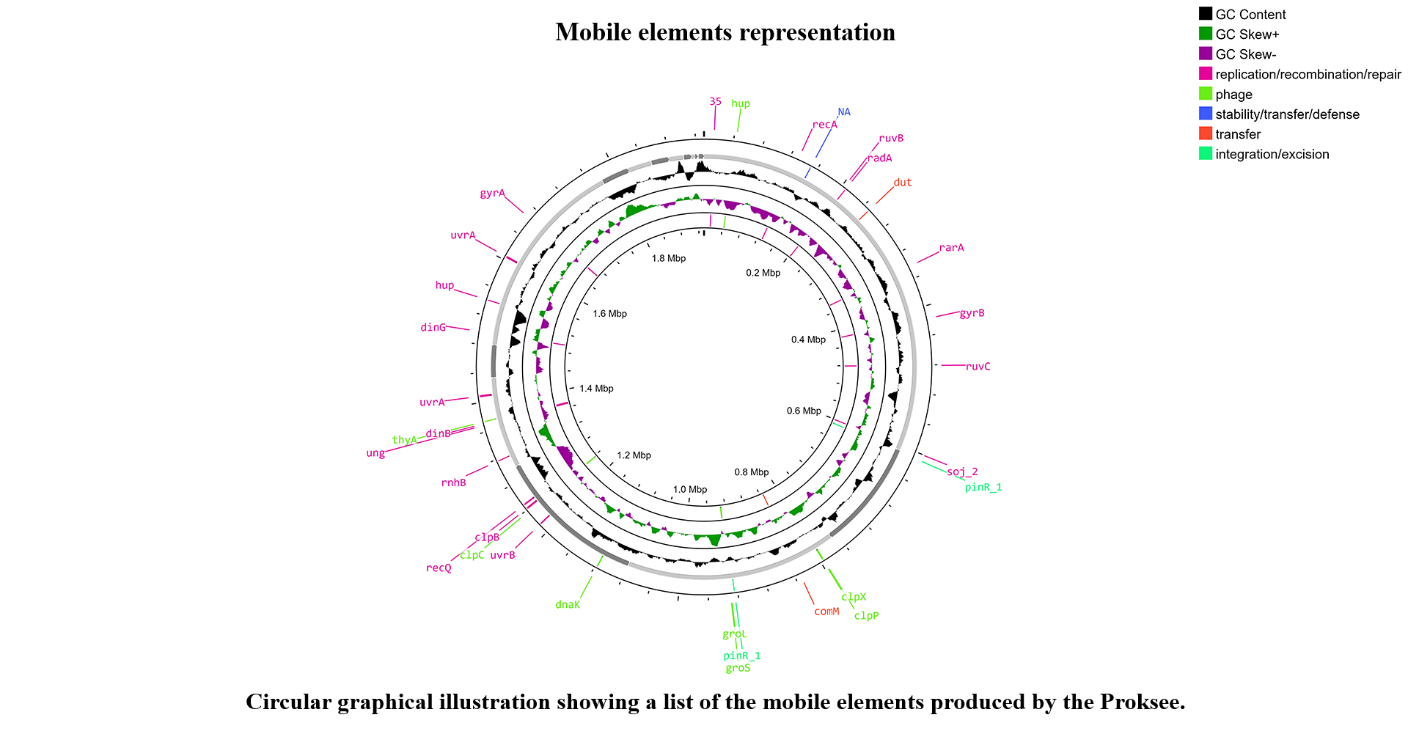


**legend:** Circular graphical illustration showing a list of the mobile elements produced by the Proksee.
